# Myocardial fibrosis is a prevalent finding in elite high-endurance athletes

**DOI:** 10.1186/1532-429X-11-S1-O68

**Published:** 2009-01-28

**Authors:** Myra S Cocker, Oliver Strohm, David J Smith, Craig Butler, Israel Belenkie, Willem Meeuwisse, Matthias G Friedrich

**Affiliations:** 1 Stephenson CMR Centre at the Libin Cardiovascular Institute, University of Calgary, Calgary, AB Canada; 2 Human Performance Lab, Faculty of Kinesiology, University of Calgary, Calgary, AB Canada; 3 Department of Cardiac Sciences at the Libin Cardiovascular Institute, University of Calgary, Calgary, AB Canada; 4 Sports Medicine Centre, University of Calgary, Calgary, AB Canada

**Keywords:** Late Gadolinium Enhancement, Myocardial Fibrosis, Elite Athlete, Late Gadolinium Enhancement Image, Cardiac Morphology

## Background

Autopsies in athletes have shown that athletes have diffuse myocardial fibrosis, and epidemiological findings place athletes at a greater relative risk of sudden death due to cardiovascular causes. Electrocardiography abnormalities are well documented in athletes, where it is widely accepted that these are characteristic of the 'athlete's heart'. Furthermore, biochemical markers of collagen breakdown and myocardial fibrosis are elevated in athletes. As such, using CMR late Gadolinium enhancement (LE) imaging, we hypothesized that fibrosis is a feature of the athlete's heart and is associated with reduced cardiac function.

## Methods

48 elite athletes (25 male, age 32 ± 13 years), and a control group of 8 healthy individuals (4 male, 31 ± 9 years) were prospectively recruited. On a 1.5 T MRI system, standard protocols for assessment for LV function and T1-weighted LE were performed.

Two experienced observers assessed LE images visually for the presence of fibrosis. The extent of fibrosis was assessed quantitatively, using semi-automated detection where regions that had signal enhancement above a threshold of 5 standard deviations from the mean signal of healthy myocardium, were considered to represent fibrosis. Areas that had a lack of contrast enhancement were used to define healthy myocardium.

Abbreviations utilized: LVEDVI – LV end-diastolic volume indexed-to-height; LVESVI – LV end-systolic volume indexed-to-height; LVSVI – LV stroke volume indexed-to-height; LVEF – LV ejection fraction

## Results

**Figure 1:**
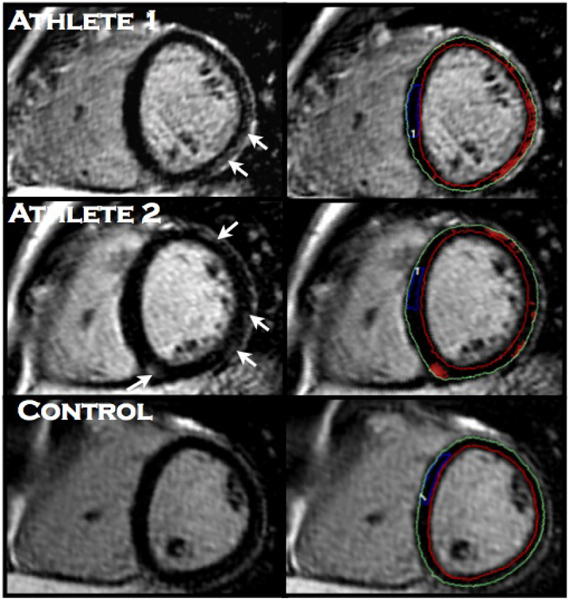
**T1-weighted late gadolinium enhancement images**. *Left panels*: Evidence of subepicardial non-ischemic myocardial fibrosis in athletes 1 and 2 (arrows), with none in healthy control. *Right panels*: Quantification of myocardial fibrosis by manually tracing a region of interest in health remote myocardium (blue outline). Automated computer thresholding set at 5 standard deviations above the mean signal intensity of remote myocardium detects fibrosis (red overlay). The detected extent of fibrosis for athlete 1 is 14.12%, 11.30% for athlete 2 and 0.20% for control.

37 of 48 (77%) athletes had non-ischemic diffuse LE, compared with 1 of 8 (13%) of controls. The extent of myocardial fibrosis in athletes who had visual evidence for LE was 10.7 ± 3.3%. Athletes with fibrosis had increased LVEDVI (117 ± 19 vs. 98 ± 15 ml/m, p < 0.05), LVESVI (44 ± 11 vs. 34 ± 10 ml/m, p < 0.05), and LVSVI (73 ± 11 vs. 64 ± 7 ml/m, p < 0.05), while LVEF (63 ± 5 vs. 66 ± 6%, p > 0.05) did not differ, when compared to those who did not have fibrosis. Figure 1.

## Conclusion

This is the first evidence of myocardial fibrosis being a prevalent finding in elite athletes, and its relation to cardiac morphology with depressed cardiac function. Long-term follow-up is required to assess the impact of fibrosis on outcome and prognosis in the 'athlete's heart'.

